# Acute Effects of Liothyronine Administration on Cardiovascular System and Energy Metabolism in Healthy Volunteers

**DOI:** 10.3389/fendo.2022.843539

**Published:** 2022-02-28

**Authors:** Shanshan Chen, George F. Wohlford, Alessandra Vecchie’, Salvatore Carbone, Sahzene Yavuz, Benjamin Van Tassell, Antonio Abbate, Francesco S. Celi

**Affiliations:** ^1^ Division of Endocrinology Diabetes and Metabolism, Virginia Commonwealth University, Richmond, VA, United States; ^2^ Department of Biostatistics, Virginia Commonwealth University, Richmond, VA, United States; ^3^ Department of Pharmacotherapy and Outcomes Sciences, Virginia Commonwealth University, Richmond, VA, United States; ^4^ Division of Cardiology, Virginia Commonwealth University, Richmond, VA, United States; ^5^ Department of Internal Medicine, ASST Sette Laghi, Varese, Italy; ^6^ Department of Kinesiology and Health Sciences, College of Humanities and Sciences, Virginia Commonwealth University, Richmond, VA, United States

**Keywords:** liothyronine, rapid effects of thyroid hormone, cardiovascular function, energy expenditure, pharmacokinetics, pharmacodynamics

## Abstract

**Context:**

The pharmacokinetics of liothyronine causes concerns for cardiovascular toxicity. While the effects of sustained increase in serum T3 concentrations are well described, little is known on the effects of acute changes in T3 concentrations due to rapid action of thyroid hormone.

**Objective:**

To assess the clinical relevance of transient increase of T3 levels on cardiovascular system and energy metabolism.

**Setting:**

Double-blind, three arms, placebo controlled, cross-over study (ClinicalTrials.gov Identifier: NCT03098433).

**Study Participants:**

Twelve volunteers (3 females, 9 males), age 27.7 ± 5.1 years.

**Intervention:**

Oral administration of liothyronine 0.7 mcg/kg, equimolar dose of levothyroxine (0.86 mcg/kg), or placebo in three identical study visits. Blood samples for total T3, free T4 were collected at times 0’, 60’ 120’ 180’ 240’. Continuous recording of heart rate, blood pressure, and hemodynamic data was performed using the volume clamp method. Resting energy expenditure was measured by indirect calorimetry. An echocardiogram was performed on each study visit at baseline and after the last blood sampling.

**Main Outcome Measures:**

Changes in cardiovascular function and energy expenditure.

**Results:**

Following the administration of liothyronine, serum T3 reached a C_max_ of 421 ± 57 ng/dL with an estimated T_max_ of 120 ± 26 minutes. No differences between study arms were observed in heart rate, blood pressure, hemodynamics parameters, energy expenditure, and in echocardiogram parameters.

**Conclusions:**

The absence of measurable rapid effects on the cardiovascular system following a high dose of liothyronine supports the rationale to perform long-term studies to assess its safety and effectiveness in patients affected by hypothyroidism.

## Introduction

The goals of the treatment of hypothyroidism are “to achieve a state of euthyroidism and normalization of the circulating levels of TSH and thyroid hormones” (1), whereby TSH is used as a powerful and reliable proxy for euthyroidism owing to its robust correlation with free T4 concentration (2). This is commonly achieved with levothyroxine (LT4) which is relatively inexpensive, available in multiple strengths and administered in single dose. This strategy relies on the conversion of the prodrug LT4 into its active metabolite T3 to correct the lack of endogenous T3 production (estimated as 15% of the total circulating pool) from the thyroid gland (3). Experimental data (4, 5) indicate that LT4 alone is not sufficient to restore tissue euthyroidism, and clinical observations (6) demonstrate that circulating levels of T3 are reduced in patients receiving LT4 therapy. Clinically, a significant percentage of patients adequately treated with LT4 complain of residual symptoms which may be attributed to hypothyroidism (7). Collectively, these observations have prompted interest in LT4/Liothyronine (synthetic T3, LT3) combination therapy or desiccated thyroid extracts as means to correct for the loss of endogenous T3, and improve symptomatology (8).

Whereas most clinical studies have showed modest to nihil improvement in symptoms and quality of life (1), a plurality of patients appeared to prefer combination therapy (9, 10). Moreover, a secondary analysis of the largest study indicated that carriers of the Ala92 allele of the type 2 deiodinase gene showed a significant improvement in quality of life (11). These observations contribute to a renewed interest in the therapeutic and pharmacologic use of LT3 (8, 12, 13), with a specific interest in developing “new well-designed adequately powered clinical trial of combination therapy” (14).

The pharmacokinetic characteristics of LT4 allow for once-daily administration. In contrast, LT3 has a short distribution half-life resulting in significant changes in serum T3 concentrations (15). Thus, a once-daily administration regimen would result in post-absorptive peaks above the normal range (15). Since extended release formulations of LT3 are not available, in LT3/LT4 combination therapy a twice daily low-dose LT3 administration is recommended to minimize fluctuations of serum T3 (13, 14). The tradeoff is a cumbersome regimen which is not convenient for a lifelong therapy, as well complicating the recruitment and retention in clinical studies (16).

**Figure 1:**
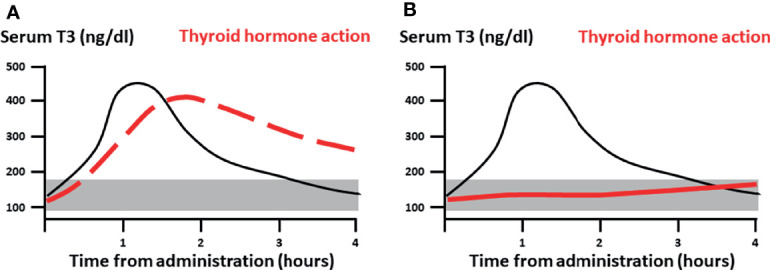
Study hypothesis. **(A)** If clinically relevant, rapid action of LT3 (dashed red line), would be measurable following a pharmacological dose at or immediately after C_max_. **(B)** Conversely, if the thyroid action is entirely attributable to genomic signaling (continuous red line) one would expect minimal measurable effects during the distribution phase with a gradual and delayed onset. Black continuous line: pharmacokinetics of single dose LT3. The shaded area represents the reference range for serum T3.

The main concern for cardiovascular complications due to transient rise in serum T3 concentrations above the normal range stems from extrapolations from chronic exposure to supraphysiologic doses (either endogenous or exogenous) of T3, and from the *in vitro* evidence of rapid, non-genomic effects of T3 in vascular endothelium and cardiomyocytes (17, 18), which would be directly exposed to fluctuations in the T3 concentrations as a result of LT3 administration. We hypothesized that if there is a clinically relevant rapid action of LT3, we would be able to detect measurable and clinically-significant, temporal changes in cardiovascular function and energy expenditure following a high, single-dose LT3 (**Figure 1**). Otherwise, from the clinical perspective, the action of LT3 would be entirely attributable to long-term transcriptional effects, and transient changes in serum T3 concentrations would not be a concern, supporting single daily administration of LT3 alone or in combination with LT4.

Here we present a pharmacokinetics-pharmacodynamics study designed to assess the clinical relevance of rapid effects of T3, with a particular focus on myocardial function, systemic vascular resistance, and energy metabolism.

## Materials and Methods

### Study Design

The study design was double blind, controlled, crossover with two active formulations (LT3 and LT4), and placebo (ClinicalTrials.gov Identifier: NCT03098433). The study was approved by the Virginia Commonwealth University IRB and all study participants provided written informed consent. All study procedures were conducted in the Clinical Research Service Unit (CRSU).

Inclusion criteria were age 18-45 years, BMI 20-30 kg/m^2^, and TSH 0.5-5.0 μIU/mL. Exclusion criteria were: thyroid autoimmunity by history or positive anti-thyroid peroxidase (TPO) antibodies; pregnancy or lactation; hypothyroidism; use of prescription drugs; diabetes mellitus; dyslipidemia; coronary artery disease; hypertension; anemia; renal insufficiency; liver disease or ALT >2.5x the upper laboratory reference limit; psychiatric conditions; tobacco use.

**Figure 2:**
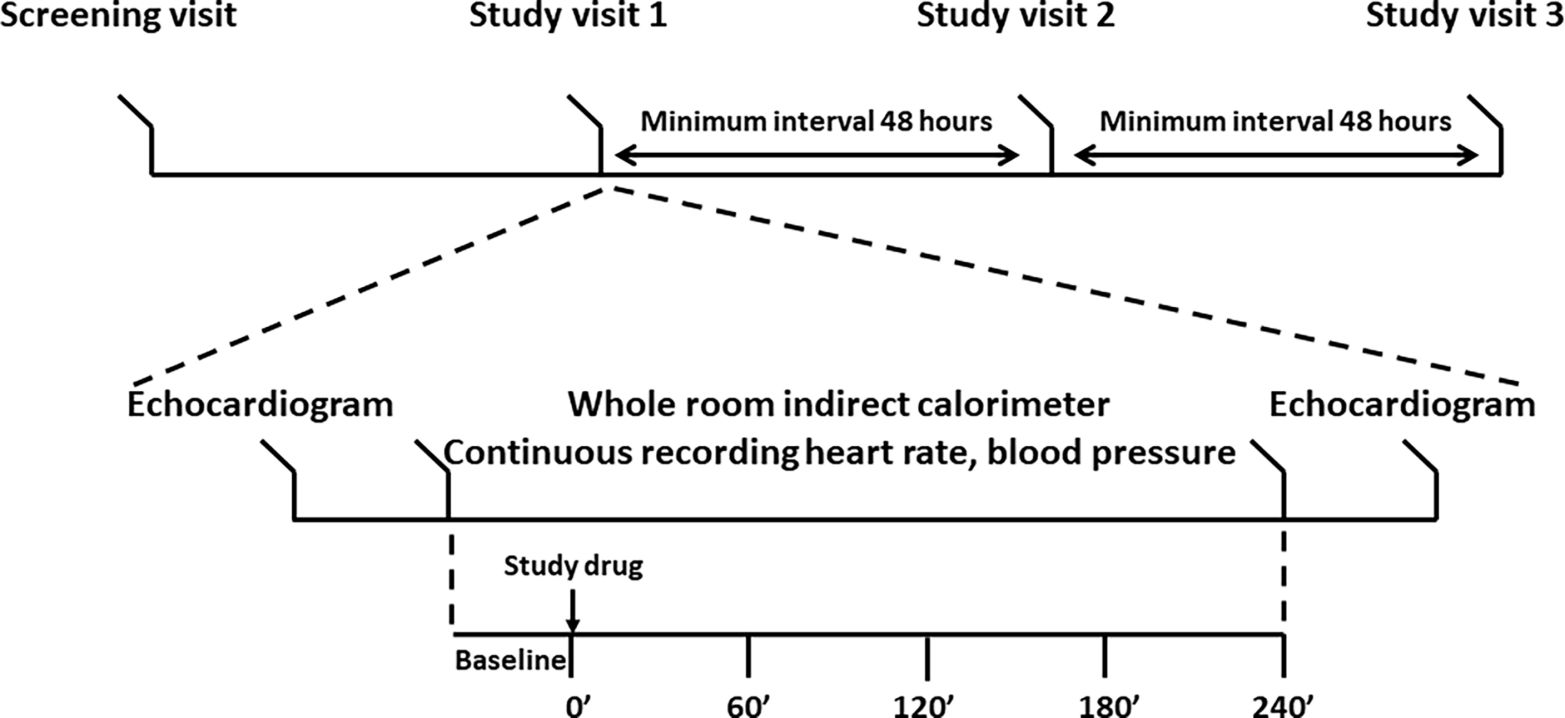
Study design. Top panel: timeline of the study. After enrollment, study volunteers underwent three identical visits each separated by at least 48 hours. Bottom panel: study procedures. An echocardiogram was performed upon arrival to the Clinical Research Services Unit and after completion of the stay in the whole room indirect calorimeter. Energy expenditure was recorded for at least 30’ before the administration of the study drug or placebo. Five blood samples were collected to record the LT4 and LT3 pharmacokinetics. Blood pressure, heart rate, and hemodynamics data were collected throughout the stay in the whole room calorimeter.

Qualifying volunteers underwent three identical study visits separated by at least 48 hours. The study scheme and procedures are reported in **Figure 2**. Study participants were instructed to refrain from strenuous exercise the day before the study. Upon arrival to the CRSU after an overnight fast, study volunteers were fitted with an i.v. cannula, vital signs were recorded, and an echocardiogram was performed. Next, the volunteers entered the whole-room indirect calorimeter (WRIC), were fitted with the Nexfin ™ pulse-wave monitor, and a 40’ baseline recording of Energy Expenditure (EE) was conducted before ingesting the study drugs or placebo. Blood samples were collected at times 0’, 60’ 120’ 180’ 240’. Immediately after the last blood draw the volunteers underwent echocardiogram, and were discharged from the CRSU. All studies were conducted in the morning, and each individual participant had their studies performed at the same time of the day. We selected a 4-hour observation window to capture any PD events occurring immediately after the C_max_ based on our knowledge of the PK characteristics of liothyronine (1).

### Drug Formulation and Dosing

Oral liquid formulation LT3 (Liotir^®^ 100 mcg/ml), LT4 (Tirosint^®^ 100 mcg/ml), and placebo were kindly donated by IBSA Institut Biochimique Lugano (Switzerland), and utilized under a (research) FDA Investigational New Drug (IND) (n.132993, Sponsor Francesco S. Celi). Study drugs were stored and dispensed by the VCU Investigational Pharmacy which performed the randomization and maintained the study blind. LT3 was administered at a weight-based dose of 0.7 mcg/kg (equivalent to a dose of 50 mcg in a 70 Kg individual), and LT4 was administered at an equimolar dose of 0.86 mcg/kg.

### Assays

Serum samples were separated on the day of the study and stored in a -80°C. Free T4, total T3 and TSH assays were processed in batch on an Abbott Architect i2000SR Immunoassay analyzer by the VCU Division of Clinical Pathology. Intra-assay variability for free T4 (reference range 0.7-1.5 ng/dL) was 2.3-3.8%; inter-assay variability was 3.6-5.2%. Intra-assay variability for total T3 (reference range 60-181 ng/dL) was 1.9-2.7%; inter-assay variability was 2.3-7.3%. Intra-assay variability for total TSH was 1.2-2.0%; inter-assay variability was 1.7-3.3%. All other assays were performed on an Abbott Architect c8000 Clinical Chemistry analyzer with the exception of TPO antibodies (LabCorp).

### Energy Expenditure

The recording was conducted in a validated, small (about 5000 liters in volume) WRIC fitted with airtight ports to allow for blood draws. This WRIC system allows for accurate and fast measure of energy expenditure (EE) with an error of ±45kcal/day (0.03 kcal/min on average). This error range means that a meaningful effect size in EE is above 0.06 kcal/min (2, 3). Throughout the recording the study participants were seated on a phlebotomy chair in resting position.

### Doppler Echocardiography

Resting transthoracic Doppler echocardiography was recorded before entering in the WRIC and immediately after the completion of the study (240’). The following parameters were recorded: left ventricular (LV) end‐diastolic and LV end‐systolic volumes, LV ejection fraction (LVEF) as a measure of systolic function; early mitral annular velocities by tissue Doppler averaged between the lateral and septal (e′) annulus and the early transmitral velocity (E) on pulsed‐wave Doppler spectra. These measures were used to calculate the E/e′ ratio, a surrogate of left ventricular filling pressures. Tricuspid Annular Plane Systolic Excursion (TAPSE) was used to assess right ventricular function (4, 5). The measurements were performed by a cardiologist (AA) blinded to the treatment on an IE33 Phillips apparatus.

### Continuous Measurements of Cardiovascular Function

Heart rate (HR), blood pressure (BP), and hemodynamic data cardiac output (CO), stroke volume (SV), and systemic vascular resistance (SVR), a proxy for endothelial vascular function, were measured with a ccNexfin system (Edwards Lifesciences Corp) (6–8) located in the WRIC. This is a non-invasive device based on the volume clamp method, which continuously measures BP by clamping the artery to a constant volume by dynamically providing equal pressure on either side of the arterial wall, while the volume is measured by a photo-plethysmograph built into a finger cuff. This device is very precise and accurate when compared with measurement of cardiac output using pulmonary artery catheter thermodilution (6, 9).

### Statistical Analysis

Pharmacokinetic (PK) parameters for LT3 following LT3 administration and LT4 following LT4 administration were estimated using each individual participant’s observed concentration data (with and without background correction) as assessed by noncompartmental analysis (NCA) using PKanalix version 2020R1 (Lixsoft^©^) software. EE, hemodynamic metrics and echocardiogram data were preprocessed in Matlab 2020a (Mathworks Inc, Natick, Massachussetts), and analyzed using mixed-effects models in R Studio (R version 3.6.3, RStudio Inc., Boston, Massachusetts). To test whether acute effects of study drugs occurred, we modeled the interaction effects of linear as well as quadratic time effects and the drugs (LT3 and LT4), and significant interaction effects. The first 20~40 minutes in the WRIC were indexed as 0 and the following minutes were indexed as [1, 2, …,240], such that the baseline EE and baseline hemodynamic data were aggregated and adjusted as intercepts in the linear mixed-effects model. The serial correlations in these time series were modeled using an autoregressive correlation structure of order 1 [i.e. AR (1)]. In the mixed-effects model for analyzing echocardiogram data, we modeled the linear change of pre- and post-study, and the interaction effects of the linear time effects and the doses (LT3 and LT4). Lastly, we used two one-sided test (TOST) procedure to detect whether the estimated fixed effects in these models are falling within regions that are equivalently to zero, given pre-defined lower and upper bounds.

## Results

### Study Population

**Figure 3:**
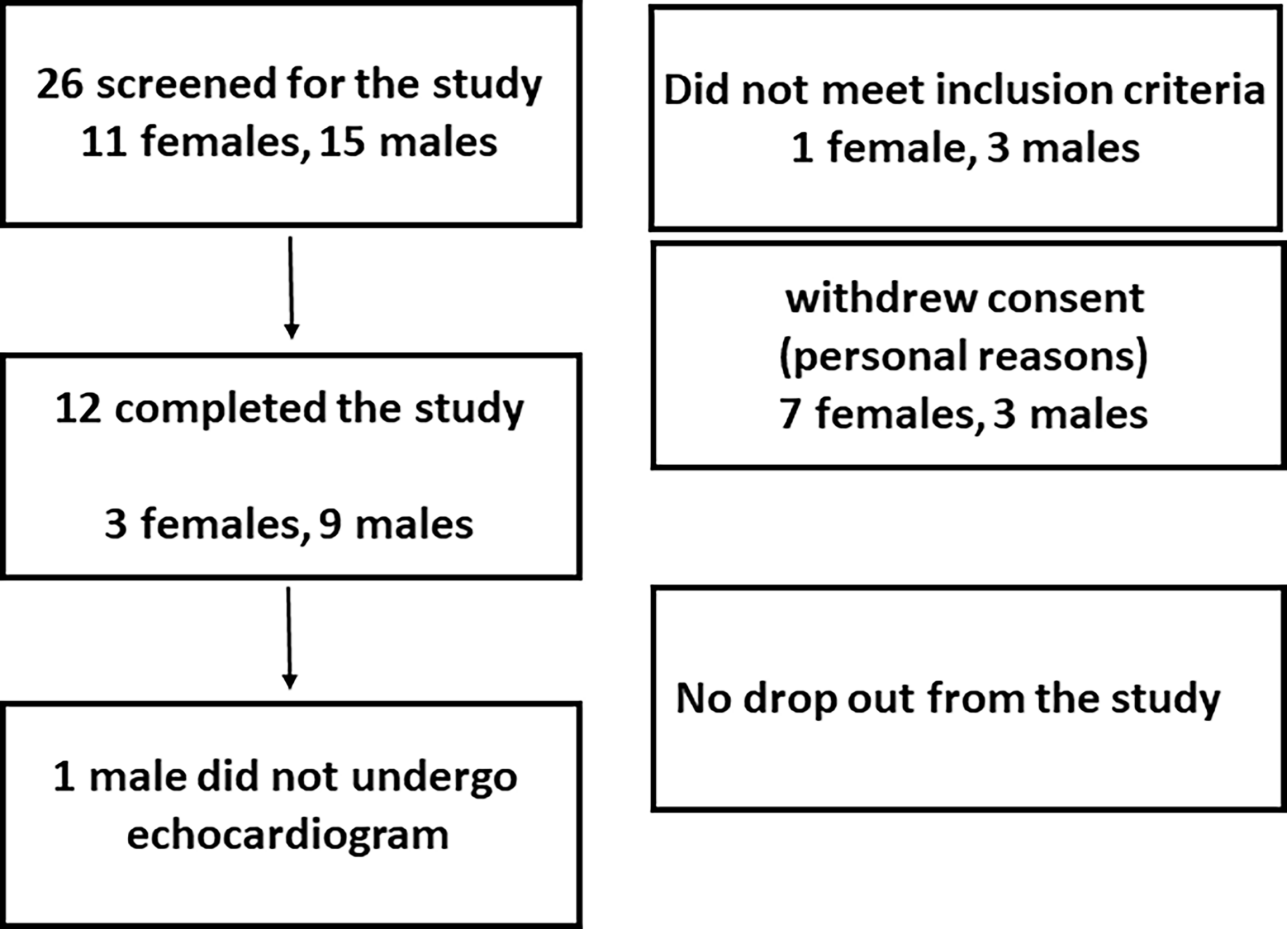
CONSORT chart.

The accrual occurred between July, 2017 and June 2019, and twelve volunteers (3 females, 9 males, age 27.7 ± 5.1 years, weight 75.0 ± 12.9 Kg) completed the study. LT3 and LT4 doses were 52.5 ± 9.0 mcg, and 64.5 ± 11.1 mcg, respectively. No adverse event was reported, and study volunteers did not report any subjective difference in well-being, anxiety, thermoregulation, or heart rate among the three interventions. The study population characteristics are reported in **Table 1**, while the recruitment details are reported in **Figure 3** (CONSORT chart).

**Table 1:** Study Participants characteristics.

	Females (3)	Males (9)	All
Age (yr)	30.3 ± 7.7	26.8 ± 4.1	27.7 ± 5.1
Weight (Kg)	61.7 ± 7.4	79.4 ± 11.3	75.0 ± 12.9
Height (cm)	168.5 ± 4.8	177.2 ± 8.7	175.0 ± 8.6
BMI (Kg/m^2^)	21.5 ± 1.8	25.2 ± 2.3	24.3 ± 2.7
Systolic BP (mmHg)	106 ± 11	121 ± 7	117 ± 10
Diastolic BP (mmHg)	61 ± 5	71 ± 6	69 ± 7
Heart rate (bpm)	71 ± 14	67 ± 10	68 ± 10
TSH (μIU/mL)	0.86 ± 0.24	1.46 ± 0.84	1.30 ± 0.77
FreeT4 (ng/dL)	1.17 ± 0.06	1.02 ± 0.07	1.05 ± 0.09
Fasting Glucose (mg/dL)	85.3 ± 5.9	89.7 ± 8.5	88.6 ± 7.9

### Pharmacokinetics

**Figure 4:**
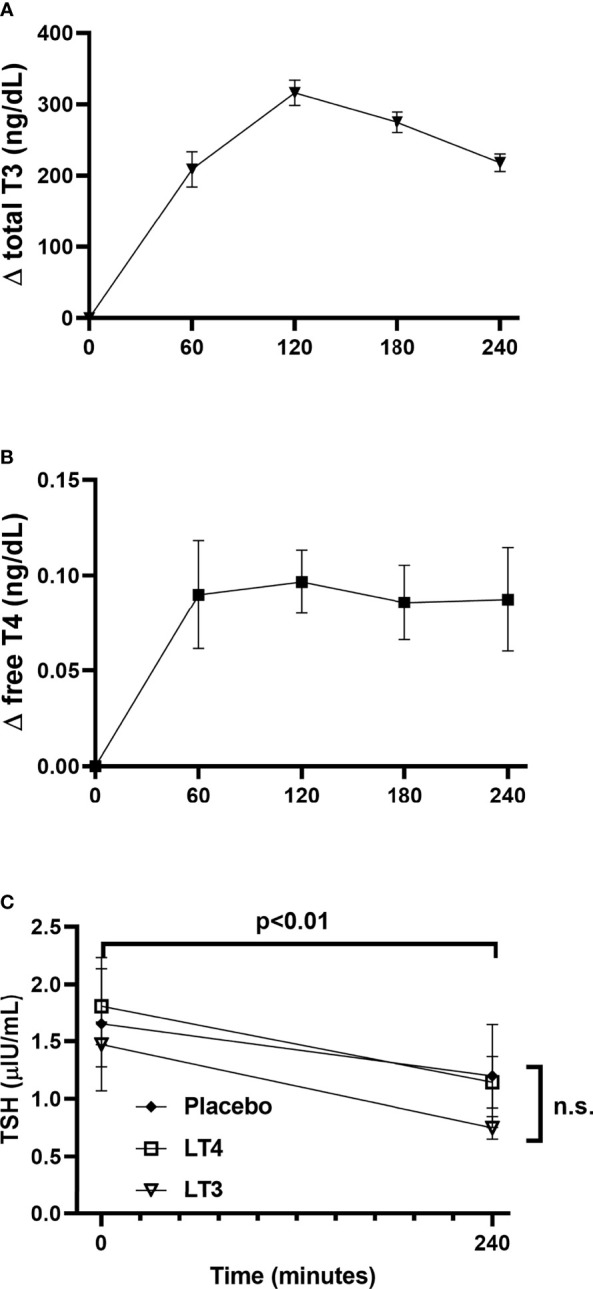
Thyroid hormone and TSH kinetics following study drugs administration. **(A)** changes in total T3 concentration from baseline following LT3 administration. **(B)** changes in free T4 concentration from baseline following LT4 administration. **(C)** TSH concentrations at baseline and 240’ following administration of LT3, LT4, and placebo. Data are presented as mean ± SEM. ns, not significant.

All the potential combinations in the randomization sequence were observed; two subjects were assigned to each combination sequence with the exception of LT4-LT3-placebo (three subjects) and LT4-placebo-LT3 (one subject). The PK parameter estimates for total T3 following oral LT3 administration and free T4 following oral LT4 administration are presented in **Table 2** and **Figure 4**. Following a single dose of LT3, the T_max_ was estimated to be 120 ± 26 minutes with a C_max_ of 328 ± 57 ng/dL when corrected for baseline. The observed clearance (assuming complete oral bioavailability) was estimated to be 45 ± 13 mL/min with an observed terminal half-life (t1/2) of 216 ± 51 minutes. The calculated AUC from baseline to 240 minutes was 54500 ± 8900 min*ng/dL. Repeating the analysis, without correcting baseline, the observed C_max_ was estimated to be 421 ± 57 ng/dL with an observed terminal t1/2 of 294 ± 69 minutes. The administration of a single dose of LT4 resulted in modest observable increases in free T4. However, when the observed concentrations were background corrected, only 2 subjects met criteria for NCA. This was attributed to the limited sampling schedule that did not capture the terminal phase. When the analysis was repeated without correction for endogenous T4, 8 of the 12 subjects data allowed for full parameter estimation by NCA. The T_max_ was estimated to be 130 ± 80 minutes and C_max_ 1.1 ± 0.15 ng/dL. The observed clearance was 27 ± 9 mL/min and the calculated AUC from baseline to 240 minutes was 246 ± 28 min*ng/dL. The reported PK parameter estimates for LT4 following LT4 administration should be considered in the setting of the limited PK sampling schedule and likely limited capture of the terminal phase.

**Table 2:** Non-compartmental analysis pharmacokinetic parameter estimates.

Pharmacokinetic Parameter	Total T3 following LT3 administration (background correction)	Total T3 following LT3 administration (without background correction)	Free T4 following T4 administration (without background correction)
N, subjects with full PK parameter estimates available	12	12	8
t_max,_ min (SD)	120 (26)	120 (26)	130 (80)
C_max,_ ng/dL (SD)	327 (57)	421 (57)	1.1 (0.15)
t_1/2_, min (SD)	216 (51)	294 (69)	1670 (630)
CL, mL/min, (SD)	45 (13)	26 (6)	2700 (940)
AUC(0-240), min • ng/dL, (SD)	54500 (8900)	76900 (8600)	246 (28)

A significant decrease in TSH from 0’ to 240’ was observed in all treatment arms (LT3 0.72 ± 0.40 LT4 0.66 ± 0.46, placebo 0.45 ± 0.49 μIU/mL, all p<0.01) (**Figure 4**). A mixed-effects model analysis indicates no significant difference in TSH decrease following LT4 or LT3 administration compared with following placebo (**Table 3**).

**Table 3:** Linear mixed-effects model for changes in TSH.

Serum TSH Level (μIU/mL)			*p*
*Predictors*	*Estimates*	*CI*	
(Intercept)	1.65	0.94 – 2.36	**<0.001**
Dose [LT3]	-0.18	-0.77 – 0.41	0.543
Dose [LT4]	0.15	-0.44 – 0.74	0.608
Post-study	-0.45	-1.04 – 0.14	0.13
Dose [LT3] * Post-study	-0.27	-1.10 – 0.56	0.519
Dose [LT4] * Post-study	-0.21	-1.04 – 0.63	0.621

Intercept captures the baseline average of TSH level pre-study on placebo days. Dose [LT3] and Dose [LT4] captures the difference of the baseline TSH on LT3 days and LT4 days, in comparison to those on placebo days. Post-study indicates the pre- and post- study change of the outcomes on placebo days. Dose [LT3] * Post-study captures differences of the pre- and post- study change between LT3 and placebo. Dose [LT4] * Post-study captures differences of the pre- and post- study change between LT4 and placebo. Numbers for the effect sizes (Estimates) and 95% confidence interval (CI) were rounded to 2 decimal places. We did not observe significant differences of pre- and post- study changes in TSH levels from either LT3 or LT4 administration in comparison to placebo administration.

Bold values indicate statistical significance.

### Physiology Parameters

Compared to placebo, no temporal change was observed in EE or substrate utilization. No significant temporal changes in HR, systolic or diastolic BP, CO or SVR were observed following the administration of the study drugs or placebo. Similarly, no differences were observed in the Doppler echocardiography studies conducted before and after administration of the study drugs or placebo. These data are reported in **Tables 4**, **5**.

**Table 4:** Results from linear mixed-effects models for changes in EE and hemodynamic metrics.

Fixed Effects	EE			Heart Rate			Cardiac Output		
	*Estimates*	*SE*	*p*	*Estimates*	*SE*	*p*	*Estimates*	*SE*	*p*
(Intercept)	1.281	0.073	**<0.001**	66.828	2.051	**<0.001**	6.427	0.348	**<0.001**
Dose [LT3]	-0.025	0.033	0.451	1.508	1.189	0.218	0.140	0.346	0.690
Dose [LT4]	0.031	0.033	0.364	1.714	1.181	0.161	0.043	0.344	0.901
Time	0.000	0.000	0.801	-0.020	0.018	0.265	0.000	0.004	0.911
Time^2^	0.000	0.000	0.906	0.000	0.000	0.168	0.000	0.000	0.870
Dose [LT3] * Time	0.000	0.000	0.813	-0.004	0.022	0.867	0.002	0.005	0.623
Dose [LT4] * Time	0.000	0.000	0.475	0.018	0.021	0.409	0.002	0.005	0.679
Dose [LT3] * Time^2^	0.000	0.000	0.976	0.000	0.000	0.676	0.000	0.000	0.704
Dose [LT4] * Time^2^	0.000	0.000	0.814	0.000	0.000	0.278	0.000	0.000	0.448
	**Stroke Volume**			**Systemic Vascular Resistance**					
	***Estimates***	***SE***	***p***	***Estimates***	***SE***	***p***			
(Intercept)	97.951	5.616	**<0.001**	1449.916	158.203	**<0.001**			
Dose [LT3]	-0.931	4.879	0.850	-108.190	185.411	0.566			
Dose [LT4]	-2.704	4.871	0.584	-28.161	183.971	0.880			
Time	0.038	0.034	0.265	2.498	2.898	0.389			
Time^2^	0.000	0.000	0.137	-0.008	0.015	0.587			
Dose [LT3] * Time	0.025	0.043	0.569	-3.311	3.340	0.322			
Dose [LT4] * Time	-0.002	0.043	0.965	-4.873	3.319	0.142			
Dose [LT3] * Time^2^	0.000	0.000	0.833	0.021	0.017	0.197			
Dose [LT4] * Time^2^	0.000	0.000	0.980	0.022	0.017	0.182			

Intercept captures the baseline average of each outcome during the first 20-40 minutes in the chamber before the dose administration of on placebo days. Dose[LT3] and Dose[LT4] captures the difference of the baseline averages on LT3 days and LT4 days, in comparison to that on placebo days. Time (Minute) and Time^2^ indicate the linear and quadratic effect of time on placebo days, capturing the temporal trends of each outcome. Dose [LT3] * Time captures the difference in linear time effects between the placebo and the LT3 dose, and Dose [LT3] * Time^2^ captures the difference in quartic time effects between the placebo and the LT3 dose. Dose [LT4] * Time captures the difference in linear time effects between the placebo and the LT4 dose, and Dose [LT4] * Time^2^ captures the difference in quartic time effects between the placebo and the LT4 dose. Numbers for the effect sizes (Estimates) and Standard Errors (SE) were rounded to 3 decimal places. The zero values indicate that there were no temporal trends observed in the time series of the outcomes on placebo days or on the active dose days. Bold values indicate statistical significance.

**Table 5:** Results from linear mixed-effects models for changes in echocardiogram outcomes.

Fixed Effects	LVEF			E			E/e’			TAPSE		
	*Estimates*	*SE*	*p*	*Estimates*	*SE*	*p*	*Estimates*	*SE*	*p*	*Estimates*	*SE*	*p*
(Intercept)	59.182	1.701	0.000	14.327	0.521	0.000	5.493	0.315	0.000	2.450	0.105	0.000
Dose [LT3]	2.000	1.604	0.219	-0.324	0.585	0.583	0.205	0.269	0.450	0.047	0.090	0.603
Dose [LT4]	0.494	1.654	0.767	0.351	0.603	0.563	0.121	0.269	0.655	-0.102	0.093	0.285
Post-study	-0.306	1.654	0.854	0.176	0.603	0.772	0.123	0.278	0.660	0.095	0.093	0.313
Dose [LT3] * Post	0.488	2.304	0.833	-0.008	0.840	0.993	-0.112	0.389	0.775	-0.122	0.127	0.344
Dose [LT4] * Post	0.906	2.359	0.703	0.089	0.860	0.918	-0.089	0.390	0.821	0.033	0.130	0.802

Intercept captures the baseline average of echocardiogram metrics pre-study on placebo days. Dose [LT3] and Dose [LT4] captures the differences of the baseline averages on LT3 days and LT4 days, in comparison to those on placebo days. Post-study indicates the pre- and post- study change of the outcomes on placebo days. Dose [LT3] * Post-study captures differences of the pre- and post- study change between LT3 and placebo. Dose [LT4] * Post-study captures differences of the pre- and post- study change between LT4 and placebo. Numbers for the effect sizes (Estimates) and standard errors (SE) were rounded to 2 decimal places. We did not observe significant differences of pre- and post- study changes in echocardiogram outcomes from either LT3 or LT4 administration in comparison to placebo administration.

### Equivalence Tests

We also tested the whether the estimated effect sizes in **Tables 4**, **5** are equivalence to zero using the TOST procedure. The p-values for rejecting that effect sizes fall beyond the bounded zero region are reported in **Tables 6**, **7** respectively. The small p-values indicate that those effect sizes are equivalent to zero, demonstrating that there was no difference in temporal trends of the physiological parameters or pre- and post- study effects of the echocardiography when comparing LT3 or LT4 against placebo.

**Table 6:** Equivalence tests (TOST procedure) of the estimated effect sizes in **Table 4**.

	EE		Heart Rate		Cardiac Output		Stroke Volume		Systemic Vascular Resistance	
	LB=-0.1	UB=0.1	LB=-5	UB =5	LB= -1	UB=1	LB=-10	UB=10	LB= -500	UB=500
Dose [LT3]	0.004	0.003	0.000	0.004	0.002	0.011	0.038	0.018	0.023	0.002
Dose [LT4]	0.004	0.002	0.000	0.005	0.003	0.005	0.074	0.008	0.009	0.004
Time	0.000	0.000	0.000	0.000	0.000	0.000	0.000	0.000	0.000	0.000
Time^2^	0.000	0.000	0.000	0.000	0.000	0.000	0.000	0.000	0.000	0.000
Dose [LT3] * Time	0.000	0.000	0.000	0.000	0.000	0.000	0.000	0.000	0.000	0.000
Dose [LT4] * Time	0.000	0.000	0.000	0.000	0.000	0.000	0.000	0.000	0.000	0.000
Dose [LT3] * Time^2^	0.000	0.000	0.000	0.000	0.000	0.000	0.000	0.000	0.000	0.000
Dose [LT4] * Time^2^	0.000	0.000	0.000	0.000	0.000	0.000	0.000	0.000	0.000	0.000

LB, lower bound; UB, upper bound. Two one-sided t-tests were conducted for each estimated fixed effects of the models presented in **Table 4**. P-values presented in this table indicate the significance of the TOST procedure, p-values < 0.05 indicate that null hypothesis that a fixed effect does not fall in the region of [LB, UB] are rejected, and we thus accept the alternative hypothesis that the fixed effect is zero.

**Table 7:** Equivalence tests (TOST procedure) of the estimated effect sizes in **Table 5**.

Predictors	LVEF		E		E/e		TAPSE	
	*LB=-5*	*UB=5*	*LB=-2*	*UB=2*	*LB=-1*	*UB=1*	*LB= -0.5*	*UB= 0.5*
Dose [LT3]	0.000	0.034	0.003	0.000	0.000	0.002	0.000	0.000
Dose [LT4]	0.001	0.004	0.000	0.004	0.000	0.001	0.000	0.000
Post-study	0.003	0.001	0.000	0.002	0.000	0.001	0.000	0.000
Dose [LT3] * Post	0.011	0.028	0.011	0.010	0.014	0.003	0.002	0.000
Dose [LT4] * Post	0.008	0.045	0.010	0.016	0.012	0.004	0.000	0.000

LB, lower bound; UB, upper bound. Two one-sided t-tests were conducted for each estimated fixed effects of the models presented in **Table 5**. P-values presented in this table indicate the significance of the TOST procedure, p-values < 0.05 indicate that null hypothesis that a fixed effect does not fall in the region of [LB, UB] are rejected, and we thus accept the alternative hypothesis that the fixed effect is zero. P-values that are less than 1e-4 are recorded as 0 in the table.

## Discussion

LT3 therapy for hypothyroidism is associated with weight loss, decreased cholesterol concentrations and a trend toward improved diastolic function (10). Very recently, similar findings on weight and lipid parameters have been reported in elderly patients affected by subclinical hypothyroidism (11). Additionally, experimental evidences suggest that low dose supplementation of LT3 improves myocardial contractility in patients with congestive heart failure (12). Moreover, a secondary analysis of the largest LT3/LT4 combination therapy study (13) showed improved quality of life in carriers of the inactivating Thr92Ala type-2 deiodinase polymorphism receiving LT3 supplements (14). Of interest, in this trial LT3 was administered in single daily dose. Since LT3 was originally approved for treatment of hypothyroidism in 1956 (15), this drug did not undergo the rigorous clinical testing currently necessary for approval and has been “grandfathered” for current use. Although the package insert recommends once-a day administration, the consensus among thyroidologists is to subdivide the dose to limit the fluctuations in serum T3 concentrations (16). Because of lack of long-term studies on its safety and effectiveness, the American Thyroid Association recommends against its use for the treatment of hypothyroidism (17). Specifically, the concerns for potential toxicity of supraphysiologic serum concentrations of T3 has hampered the use of LT3 alone or LT3/LT4 combination therapy in patients with hypothyroidism.

LT3 is promptly absorbed following oral administration, and doses as low as 0.25 mcg/Kg result in increase in T3 serum concentration well above the upper limit of reference, raising the concern for cardiovascular toxicity. Rapid, non-genomic actions of thyroid hormone have been demonstrated *in vitro* in endothelial vascular cells (18), providing additional rationale for the concern for toxicity due to the exposure of the vasculature to transient increase in T3 serum concentrations following LT3 administration. The interaction between T3 and integrins results in the production of nitric oxide, promoting vasodilation (19, 20). Despite these laboratory-based data, the clinical relevance of rapid effects of thyroid hormone is not clear. Studies performed in *ex vivo* models of vasculature contractility are conflicting: Gachkar and colleagues demonstrated a rapid response to physiologic concentrations of T3, and a decreased response to both hyper- and hypothyroid concentrations, not mediated by AKT, ERK or AMPK (21). More recently, other authors demonstrated an increase in vasodilation following exposure to T3, mediated by PI3K pathway and thyroid hormone receptor alpha (22). Experiments conducted in an animal model of hypothyroidism indicate that exposure to high dose LT3 generates a measurable transcriptional effect within 30’ reaching a maximum effect after 6 hours (23). Clinically, the reports of toxicity secondary to trauma with release of thyroid hormone from the gland, or following acute poisoning indicate a significant lag time (24, 25), suggesting that the majority of the harm caused by thyrotoxicosis is ascribable to transcriptional effects of thyroid hormone. It is possible that the discrepancies between laboratory-based experiments and *in vivo* observations could be explained by the dynamic of acute exposure (seconds) to thyroid hormone vs. the much slower changes in thyroid hormone concentrations following oral administration.

We speculated that, if rapid effects of T3 were of clinical relevance, transient exposure to T3 concentrations above normal range would result in measurable and potentially clinically significant changes in the cardiovascular system and energy metabolism owing to the exquisite sensitivity of these end-organ targets to the action of thyroid hormone (26, 27). Moreover, since the endothelial vascular cells are exposed to changes in T3 concentrations, measurement of their function by assessing systemic vascular resistance would represent an ideal readout to assess acute effects of supraphysiologic doses of LT3.

To test this hypothesis, we conducted a pharmacokinetics/pharmacodynamic study directed to these targets of the hormonal action. In this study we chose to investigate a short timeframe based on our knowledge of liothyronine PK (1), aiming to capture events occurring at and immediately after the C_max_, i.e. at the time of maximal exposure of endothelial and myocardial cells to the peak serum T3 concentrations. We used a pharmacologic dose which would produce peak T3 concentration well above the upper reference range. To control for potential non-specificity in the nongenomic effects of thyroid hormone, in addition to placebo we included a study arm of equimolar LT4 dose as additional control.

By using liquid formulations of LT3 and LT4 we were able to titrate the doses to adjust for the participants’ weight, further increasing the internal validity of the study. When compared to our previous observations on tablet formulation (1), liquid LT3 showed a similar T_max_, while, as expected, the C_max_ was much greater due to the higher LT3 dose employed in this study. The decrease in TSH from baseline to the end of the study, which is consistent with the hormone biorhythm (28) and possibly amplified by the prolonged fast, was not different among the active drugs and placebo.

Our data indicate no significant differences or appreciable trend during our observation in the all the study endpoints despite achieving clearly supraphysiologic serum T3 concentrations. Specifically, during the four hours following the administration of LT3, we did not observe any change in HR or BP, or estimates of CO and SVR. Moreover, we independently assessed the acute effects of LT3 on the myocardium by performing a Doppler echocardiography study before and after each of the studies. Similar to the ccNexfin continuous recordings, we observed no measurable effects on myocardial contractility. Finally, no differences were observed in energy metabolism measured by the WRIC.

Our data are consistent with the observations of Jonklaas et al. that showed significant increase in heart rate and blood pressure only after five hours from the administration of a pharmacologic dose of LT3 (29). Of note, in their study, the subjects were given lunch at 4-hour, which *per se* may have resulted in post-prandial HR elevation (30); thus, the true cardiovascular response beyond 4 hours without confounding factors remains to be captured.

Collectively, the results of our study indicate that a single administration of LT3, sufficient to transiently increase the T3 concentration well above the range of reference, does not result in significant signal in the tissues which are most sensitive to acute effects of T3, and are immediately exposed to the peak in T3 concentration. These findings appear to negate clinical relevance to the rapid effects of T3 which have been observed *in vitro*. The clinical implications are intriguing, since one could speculate that these results provide the rationale to consider the use of single administration of LT3, rather than trying to reach stable serum concentrations by extended release formulations or by multiple daily administrations regimens. Conversely, we want to affirm that absent an empirical demonstration of effectiveness and safety of once daily administration regimen, this interpretation of the data should not translate in clinical practice.

Strengths of our study reside in the rigorous study design, use of liquid formulation of the drug which allowed for a precise, weight-based dosing, and the use of state-of-the-art techniques to assess the cardiovascular system and energy metabolism. The continuous recording of BP, endothelial vascular function and EE allowed us to measure even subtle changes which would not be captured by single timepoint observations. Finally, we included a treatment arm of LT4 to assess for potential non ligand-specific acute effects of thyroid hormone. To the best of our knowledge, no study has investigated the rapid effects of thyroid hormone in such details.

The relatively small sample size, non-unusual in phase I-II studies, represents a limitation. However, the absence of trends in the physiological variables indicates that it is extremely unlikely our negative findings are due to type-2 error. Some volunteers had a short, 48-hour interval between the studies raising the potential concern for carryover effects. Given the lack of measurable effects and intraindividual differences of baseline (before administration of the study drugs) data, we do not believe that this is a cofounding factor. By design, the observation was limited to a timeframe where maximum variation in serum T3 concentrations can be observed, therefore late events would not be captured. Lastly, the translational value of observation obtained in healthy individuals to patients affected by hypothyroidism, in particular elderly and with co-morbidities is unknown, and we cannot rule out the possibility that acute effects may occur in patients with lower serum T3 levels.

In conclusion, in healthy individuals a single administration of LT3, able to rapidly increase the serum T3 concentration above normal range does not result in measurable changes in target organ-systems attributable to the rapid thyroid hormone action. The data support the rationale to explore the use of LT3 in single dose aiming to achieve sustained increase in tissue concentrations of T3, reducing the concern for fluctuations in serum concentrations. On the other hand, patients who have longstanding significant hypothyroidism may experience paradoxical response, and T3-based therapy should be started after achieving a euthyroid state by LT4, and residual symptoms are still present. Long-term studies in patients affected by hypothyroidism are necessary to assess the safety and effectiveness of single dose LT3 or LT3/LT4 combination therapy in patients affected by hypothyroidism.

## Data Availability Statement

The raw data supporting the conclusions of this article will be made available by the authors, without undue reservation.

## Ethics Statement

The studies involving human participants were reviewed and approved by VCU IRB. The patients/participants provided their written informed consent to participate in this study.

## Author Contributions

SCh contributed to the study design, analyzed energy expenditure data, performed statistical analysis and contributed to the interpretation, and contributed to the initial draft and final writing of the manuscript. GFW performed pharmacokinetics analysis and contributed to the writing of the manuscript. AV recorded and analyzed echocardiography data. SCa recorded and analyzed hemodynamics data. SY contributed to the study design, clinical assessment of study patients, and contributed to the editing of the manuscript. BVT contributed to the study design and interpretation of the pharmacokinetics data. AA contributed to the study design and interpretation of the echocardiography data. FSC designed the study, supervised the analysis and interpretation of the data, and wrote and edited the manuscript. All authors contributed to the article and approved the submitted version.

## Funding

SCa is supported by a Career Development Award 19CDA34660318 from the American Heart Association and by the Clinical and Translational Science Awards Program UL1TR002649 from National Institutes of Health to Virginia Commonwealth University. FSC is supported by the NIH-NIDDK grant 1 R21 DK122310-01A1.

## Author Disclaimer

Its contents are solely the responsibility of the authors and do not necessarily represent official views of the National Center for Advancing Translational Sciences or the National Institutes of Health.

## Conflict of Interest

The Division of Endocrinology, Diabetes and Metabolism of Virginia Commonwealth University has received an unrestricted grant from IBSA Institut Biochimique, Lugano Switzerland. Liquid formulations of LT3, LT4, and placebo were a kind gift of IBSA Institut Biochimique Lugano (Switzerland). IBSA was not part of the study design and had no access to the data prior to the publication. FC has served as consultant for IBSA, Acella and Kashiv (now merged with Amneal).

The remaining authors declare that the research was conducted in the absence of any commercial or financial relationships that could be construed as a potential conflict of interest.

## Publisher’s Note

All claims expressed in this article are solely those of the authors and do not necessarily represent those of their affiliated organizations, or those of the publisher, the editors and the reviewers. Any product that may be evaluated in this article, or claim that may be made by its manufacturer, is not guaranteed or endorsed by the publisher.
